# Insulin Resistance in Human Hippocampal Pyramidal Neurons Correlates with Age in Cognitively Normal and Alzheimer's Disease Patients

**DOI:** 10.1002/alz70855_106242

**Published:** 2025-12-24

**Authors:** Xuelin Gu, Tim Distel, Konrad Talbot

**Affiliations:** ^1^ Loma Linda University, Loma Linda, CA, USA

## Abstract

**Background:**

The pathogenesis of Alzheimer's disease (AD) is not fully understood, with evidence that β‐amyloid accumulates in the brain before hyperphosphorylated tau aggregates into neurofibrillary tangles. In addition to β‐amyloid and phospho‐tau, brain insulin resistance is a common feature of AD dementia (ADd), with antidiabetics like semaglutide currently in phase 3 clinical trials for AD. While a clear biomarker for brain insulin resistance has yet to be recognized, some studies have identified elevated insulin receptor substrate‐1 phosphorylated at serine‐616 (IRS‐1^pS616^) in ADd, specifically in cytoplasmic microdomains in neurons. Here, we quantified IRS‐1^pS616^ in pyramidal neurons of hippocampal CA1 in cognitively normal and ADd cases using artificial intelligence‐based analysis of digital pathology to identify correlations between ADd pathologies and subject demographics including age.

**Methods:**

We conducted immunohistochemical studies on fixed hippocampal sections from 217 age‐ and sex‐matched cases: 68 non‐cognitively impaired (NCI), 13 preclinical (PrCl; i.e., NCI but A+T+), 24 non‐amnestic mild cognitively impaired (naMCI), 32 amnestic MCI (aMCI), and 80 ADd from 3 brain banks (Banner Research Institute, University of Pennsylvania, and Rush University) for β‐amyloid (NAB228), phospho‐tau (AT8), and IRS‐1^pS616^ (44‐550G). Immunoreactive cells and plaques were quantified using supervised deep‐learning U‐Net neural networks with additional morphology‐based criterions.

**Results:**

The density of hippocampal CA1 pyramidal neurons with IRS‐1^pS616^ pathology were low in cognitively normal post‐mortem tissue and increased with age while the same pathology was elevated in ADd and decreased with age. No significant correlations with age were identified in PrCl and MCI groups. Furthermore, IRS‐1^pS616^ pathology was positively correlated with β‐amyloid pathology, phospho‐tau pathology, and Braak staging. Coverage of IRS‐1^pS616^ plaque‐like structures was also positively correlated with β‐amyloid plaque burden in non‐sequential hippocampal tissue. No significant differences were found with ADd pathologies between ApoE genotypes.

**Conclusion:**

These results suggest that brain insulin resistance increases with age, before peaking in ADd and decreasing as neurodegeneration proliferates. The correlations between IRS‐1^pS616^, β‐amyloid, and phospho‐tau pathologies also suggest that accumulation of cytoplasmic IRS‐1^pS616^ in pyramidal neurons is involved in ADd pathogenesis. Further study may provide evidence towards a mechanism of action for insulin resistance therapies in AD with IRS‐1^pS616^ as a biomarker of brain insulin resistance.

